# Comparing strategies for United States veterans' mortality ascertainment

**DOI:** 10.1186/1478-7954-3-2

**Published:** 2005-02-24

**Authors:** Karl A Lorenz, Steven M Asch, Elizabeth M Yano, Mingming Wang, Lisa V Rubenstein

**Affiliations:** 1 VA Greater Los Angeles Healthcare System, Los Angeles CA, USA; 2 Geffen School of Medicine at UCLA, Los Angeles CA, USA; 3 RAND, Santa Monica CA, USA; 4 Department of Health Services, UCLA School of Public Health, Los Angeles CA, USA

## Abstract

**Background:**

We aimed to determine optimal strategies for complete mortality ascertainment comparing death certificates and United States (US) Veterans Administration (VA) records.

**Methods:**

We constructed a cohort of California veterans who died in fiscal year (FY) 2000 and used VA services the year before death. We determined decedent status using California death certificates linked to VA utilization data and the VA Beneficiary Identification and Records Locator System (BIRLS) death file. We compared the characteristics of decedents who would not have been identified by either single source (e.g., VA BIRLS alone or California death certificates alone) with the rest of the cohort.

**Results:**

A total of 8,813 veteran decedents were identified from both VA decedent files and death certificates. Of all decedents, 5,698 / 8,813 (65%) veterans were identified in both source files, but 2,426 / 8,813 (28%) decedents were not identified in VA BIRLS, and 689 / 8,813 (8%) were not identified in death certificates. Compared to the rest of the cohort, decedents whose mortality status was ascertained through either single source differed by race / ethnicity, marital status, and California residence. Clinically, veterans identified from either single source had less comorbidity and were less likely to have been users of VA inpatient or long term care, but equally or more likely to have been users of VA outpatient services.

**Conclusion:**

As single sources, VA decedent files and death certificates each provided an incomplete record, and death ascertainment was improved by using both source files. Potential bias may vary depending on analytic interest.

## Introduction

Clinicians, healthcare administrators, researchers, regulators and policymakers are concerned with optimizing mortality ascertainment using administrative data. In addition to its clinical importance, mortality informs program planning, quality assessment and improvement, and public reporting [1-8]. Veterans are an important, vulnerable population in which mortality has been examined as a function of race / ethnicity, service characteristics, access, and quality of care. Valid, complete reporting is critical to the success of such endeavors, and limitations in using death certificates have been acknowledged [9,10], although VA mortality data is generally regarded as accurate [11-15]. To understand the limitations of single source ascertainment, we described decedents who would not have been identified by a strategy using either VA decedent files alone or death certificates alone. We compared cases that would have been missed using either single source with the rest of the cohort based on their demographic and clinical attributes and the settings in which they received care.

## Methods

In order to evaluate the implications for improving veterans' end-of-life care, we constructed a population-based decedent cohort [16]. For such purposes, it is particularly important to understand whether death was recorded elsewhere for veterans who were under VA care since the VA system may be responsible for much of their end-of-life care even if they do not die while receiving health care in a VA facility.

### Data Sources

The VA Beneficiary Identification and Records Locator System (BIRLS) contains records of all beneficiaries including veterans whose survivors applied for burial benefits. It includes records of discharged military veterans post-1973 and recipients of Medals of Honor and VA education benefits. After submission to the Veterans Benefits Administration (VBA), deaths are recorded in the BIRLS Death File. A submission to the VBA is typically triggered by a family claim for death benefits (e.g. burial assistance, pension) [17-19]. The VA maintains a National Patient Care Database (NPCD) that contains a record of Social Security Number (SSN) linked VA and contracted health services provided to all veterans [17-19]. Death certificates are required for burial in California and are available for public use [20].

We first identified 345,380 decedent veterans who died during FY2000 (30 September 1999 – 1 October 2000) from the BIRLS Death File. We used SSNs to link cases to VA NPCD outpatient, inpatient, or long term care records restricted to recipients of any VA services in California within 12 months of death. We extracted records including any inpatient or long term care admission, or outpatient encounters. Veterans who entered the cohort on the basis of using outpatient services were required to have at least one clinical encounter (e.g., other than laboratory, radiology, or administrative).

In addition, we used California death certificates as second source to identify decedent veterans by linking SSNs from death certificates directly to VA utilization files. California death certificates contained 462,561 records for calendar years 1999 and 2000, and we primarily matched decedents identified through death certificates to BIRLS by SSN. We manually inspected matches on SSN only and we also examined matches on criteria other than SSN (e.g. last name, first name, date of birth, date of death). Additional cases we accepted after manual inspection involved transpositions of one and rarely more than one SSN digit but agreement in other fields. Thus, the cohort included recipients of VA clinical services verified as deceased based on either BIRLS or death certificates, and all cases were linked to VA utilization files by SSN.

In the final decedent cohort, we excluded cases of non-veterans receiving care at VA facilities by examining indicators of veteran status associated with visits. The VA assigns specific codes to non-veterans rendered care for various reasons (e.g., emergency or charitable care). We also considered the possibility of erroneous decedent status by looking for evidence of healthcare utilization during the 12 months after death. We excluded cases with evidence of utilization more than one month after the date of death.

### Variables and Analysis

We used VA encounters and ICD-9-CM codes to demographically (e.g., age, gender, marital status, state of residence, and race / ethnicity) and clinically characterize decedents [21-26]. We identified veterans with any visit or admission for congestive heart failure (CHF), ICD-9-CM 398.91, 402.x1, 404.x1, 404.x3 428.x excluding procedures, chronic obstructive lung disease (COPD), ICD-9-CM 491–492.x, 494.x, 496, end-stage liver disease (ESLD), ICD-9-CM 571.2–571.9,572.2–572.8, dementia, ICD-9-CM 046.1, 290.0–290.43, 331.0–331.7, 333.4, 438.0, and malignant neoplasia, ICD-9-CM 140.0–208.9 [25]. To identify end-stage renal disease (ESRD), we used procedure and clinical stop codes that identify the type of care received (e.g., dialysis) [26]. We developed a complexity index of co-morbidity based on a simple count of advanced illnesses.

To understand the limitations of single source mortality ascertainment, we described decedents who would not have been identified by a strategy using either death certificates alone or VA decedent files alone. We compared these cases with the rest of the cohort based on their demographic and clinical attributes and the settings in which they received care. Based on distributions, we used Wilcoxon tests for continuous and chi-square tests for categorical variables.

## Results

**Figure 1:**
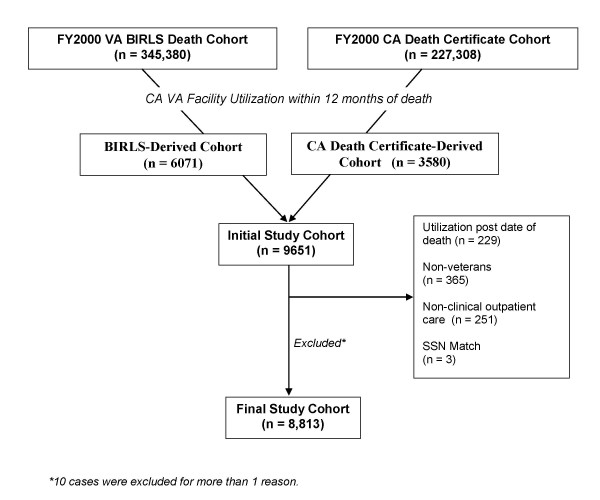
Cohort Development

From 345,380 deaths during the period 30 September 1999 to 1 October 2000 identified in BIRLS, we distinguished 6,071 decedents who were users of VA inpatient, outpatient, or long term care services in California. California death certificates included 227,308 deaths during the same period, including 3,580 additional users of VA inpatient, outpatient, or long term care services in California. Using SSN and other identifiers to match decedent cases to VA utilization data, we excluded non-veterans (n = 365), users of only non-clinical care such as laboratory tests (n = 251), those possibly alive based on subsequent VA encounter data (n = 229), and 3 cases for other reasons. Of the final cohort of 8,813 veteran decedents, 5,698 (65%) cases were identified in both source files, while 689 (8%) were only identified in VA decedent files, and 2,426 (28%) additional cases were only identified through death certificates (Figure 1).

We examined potential biases associated with veteran decedents missed by either single source of mortality ascertainment (e.g., VA BIRLS or California death certificates). Ninety-nine percent of decedents missed by using VA data alone were California residents (vs. 92% of the remainder cohort, p < 0.001); whereas, 62% of those missed by using death certificates alone were out-of-state residents (vs. 1% of the remainder cohort, p < 0.001). Relatively fewer veterans of white or black ethnicity and relatively more veterans of missing ethnicity were represented among decedents missed by either single source strategy. The proportion of married or previously married veterans was higher and single or missing marital status lower among those decedents missed using only BIRLS, and relative proportions were reversed for a strategy using only death certificates.

Decedents missed by either single source approach were less likely to have been diagnosed with an advanced chronic illness than the identified cohort. Veteran decedents missed by using only BIRLS were less likely to be diagnosed with any condition except HIV and dementia, and those missed by using death certificates alone were less likely to be diagnosed with any condition except HIV. With a BIRLS only approach, 37% of missing cases vs. 35% of the remainder cohort (p < 0.001) had no diagnosed chronic illness (death certificate only approach; 69% vs. 32%, p < 0.001). Veteran decedents missed by either single source approach were equally or more likely to have been users of the outpatient setting, but missed cases were less likely to have been users of inpatient healthcare settings (Table 1).

**Table 1:** Potential Bias Associated with Alternative Strategies For Veterans' Mortality Ascertainment *

	**BIRLS Only Strategy**			**Death Certificate Only Strategy**		
		
	Cases identified by BIRLS	Additional cases identified by death certificates	P-value	Cases identified by death certificates	Additional cases identified by BIRLS	P-value
Number of cases	6,387	2,426		8,124	689	

Age (years)	70.86	71.15	0.8253	70.95	70.79	0.6891

Gender						
Male	98	97	0.2733	98	98	0.8662

Race / Ethnicity						
White	57	54		58	31	<0.001
Black	12	8		11	6	
Hispanic	5	5		5	1	
Other	2	2		2	1	
Missing	24	31	<0.001	23	61	

Marital Status						
Married	46	49		47	45	0.0028
Single	16	13		15	16	
Divorced	23	24		23	23	
Widowed	11	12		11	11	
Missing	4	2	0.0002	3	6	

State of Residence						
California	92	99		99	38	
Non-California	8	1	<0.001	1	62	<0.001

Diagnosis						
Cancer	35	32	0.0426	35	17	<0.001
CHF	22	19	0.0175	22	7	<0.001
COPD	28	24	0.0002	28	11	<0.001
ESLD	6	4	0.0327	6	3	0.0010
ESRD	3	1	<0.0001	3	0	0.001
Dementia	11	11	0.9656	11	3	<0.001
HIV	1	1	0.1586	1	0	0.0621

Complexity Index						
0	35	37		32	69	
1	35	39		38	22	
2	22	18		22	6	
3	7	5		7	2	
4	1	1	<0.0001	1	0	<0.001

Site of Utilization						
Any inpatient						
Any long term	45	29	<0.0001	42	21	<0.001
care	20	12	<0.0001	19	7	<0.001
Any outpatient	95	96	0.0225	95	96	0.1218

*Findings are expressed as proportions unless otherwise identified. P-values reflect Wilcoxon two-sided probabilities for continuous variables and chi-square for categorical variables. Categorical tests reflect tests for differences including missing.

## Discussion

Veterans' mortality ascertainment was significantly improved by using both VA and death certificates as source files. Our findings indicate that either single source approach for mortality ascertainment may misrepresent veteran mortality based on comparisons of race / ethnicity, marital status, severity of illness, and settings of care. Diagnoses associated with serious medical co-morbidity and the likelihood of receiving any inpatient services (e.g. hospital or long term care) were both significantly lower among veterans missed by either single source approach.

Our findings are consistent with Washington State where the deaths of 25% of 533 veterans who only used outpatient services were only identified with death certificates, and 5% were only identified in BIRLS. [9] Using BIRLS only for mortality determination, it is unclear why generally healthier, primarily outpatient users are less likely to be noted. Death notification is typically triggered by benefit claims (e.g., burial assistance, pension and related benefits). Affluent veterans whose families might be less likely to file benefit claims were drawn to the VA recently [27]. However, poverty or low social support might also make it harder to file claims. On the other hand, a death certificate only approach to ascertainment misses relatively fewer non-resident veterans. Such veterans may be homeless or mobile, retired veterans, and they may seek care transiently in California, or their deaths may be recorded elsewhere.

One limitation of our study is that we did not identify cases that were only decedents by virtue of VA utilization files alone rather than BIRLS, although Dominitz, et. al., identified only 2.7% of deaths this way [11]. We did not compare VA files or death certificates to the National Death Index (NDI), as have previous studies that have used the NDI as a gold standard. The NDI is a central data repository of state vital statistics that is often used as a gold standard in US mortality studies [28]. We report findings for only one state, but given similar findings in Washington State, it would be helpful to determine if this is a national issue or there are particular state issues related to BIRLS death file agreement, or concerns related to veteran morality ascertainment with California death certificates.

## Conclusion

Researchers, managers, and policy makers should understand the limitations of sources of mortality ascertainment. The relationship of missing data to bias is related somewhat to how "missingness" is distributed by the outcome of interest. Our findings suggest these concerns may be relatively more important for studies involving veterans and racial-ethnic disparities, co-morbidity, certain disease comparisons, or settings of care. Additional study is needed to compare BIRLS, death certificates, and the NDI for mortality ascertainment in veterans. If our findings are confirmed, the VA may need to consider improving its system for mortality ascertainment through routine linkages to national mortality data. Studies of end-of-life care using decedent cohorts need to pay particular attention to the incompleteness of VA data as the sole source of mortality information.

## List of Abbreviations Used

VA, Veterans Administration; FY, fiscal year; BIRLS, Beneficiary Identification and Records Locator System; NPCD, National Patient Care Database; SSN, Social Security Number; CHF, congestive heart failure; COPD, chronic obstructive lung disease; ESLD, end-stage liver disease; ESRD, end-stage renal disease.

## Competing interests

The author(s) declare that they have no competing interests.

## Authors' contributions

KL originated and oversaw all aspects of the conception, design, analysis, and publication of the study. SA, LR, and EY contributed to conception, design, and analysis. MW contributed to analysis and is responsible for programming. All authors reviewed and approved of the manuscript.
